# Supplementary material for: Short-Chain Fatty Acids Modulate Permeability, Motility and Gene Expression in the Porcine Fetal Jejunum Ex Vivo
Source: Nutrients. 2022 Jun 17;14(12):2524. doi: 10.3390/nu14122524 (PMC9230976; doi:10.3390/nu14122524)
Supplement: Supplementary file 1 [file nutrients-14-02524-s001.zip › nutrients-1733973-supplementary.pdf]

nutrients

MDPI

Supplementary Material

Short-Chain Fatty Acids Modulate Permeability, Motility and Gene Expression in the Porcine Fetal Jejunum Ex Vivo

Barbara U. Metzler-Zebeli[1,2*], Simone Koger[2,3], Suchitra Sharma[2,3], Arife Sener-Aydemir[2,3], Ursula Ruczizka[4,5], Heinrich Kreutzmann[4], Andrea Ladinig[4]

[1] Unit of Nutritional Physiology, Department of Biomedical Sciences, University of Veterinary Medicine Vienna, 1210 Vienna, Austria; barbara.metzler@vetmeduni.ac.at
[2] Christian-Doppler Laboratory for Innovative Gut Health Concepts of Livestock, University of Veterinary Medicine Vienna, 1210 Vienna, Austria
[3] Institute of Animal Nutrition and Functional Plant Compounds, Department of Farm Animal and Veterinary Public Health, University of Veterinary Medicine Vienna, 1210 Vienna, Austria; simone.koger@vetmeduni.ac.at; suchitra.sharma@vetmeduni.ac.at; arife.sener@vetmeduni.ac.at
[4] University Clinic for Swine, Department of Farm Animal and Veterinary Public Health, University of Veterinary Medicine Vienna, 1210 Vienna, Austria; heinrich.kreutzmann@vetmeduni.ac.at; andrea.ladinig@vetmeduni.ac.at
[5] Association for Sustainable Animal Husbandry in Austria (NTÖ) – Project Animal Health Austria, 1200 Vienna, Austria; ruczizka@nutztier.at
* Correspondence: barbara.metzler@vetmeduni.ac.at.

Citation: Metzler-Zebeli, B.U.; Koger, S.; Sharma, S.; Sener-Aydemir, A.; Ruczizka, U.; Kreutzmann, H.; Ladinig, A. Short-Chain Fatty Acids Modulate Permeability, Motility and Gene Expression in the Porcine Fetal Jejunum Ex Vivo. *Nutrients* **2022**, *14*, 2524. https://doi.org/10.3390/nu14122524

Academic Editor: Jamileh Movassat

Received: 4 May 2022
Accepted: 11 June 2022
Published: 17 June 2022

Publisher's Note: MDPI stays neutral with regard to jurisdictional claims in published maps and institutional affiliations.

Copyright: © 2022 by the authors. Submitted for possible open access publication under the terms and conditions of the Creative Commons Attribution (CC BY) license (https://creativecommons.org/licenses/by/4.0/).

Table S1. Oligonucleotide primers for target and housekeeping genes [20,21].

Gene	Gene-Name	Accession number		Oligonucleotide sequence (5' to 3')	Amplicon Size	Efficiency(%)	Reference
				Reference genes			
ACTG	Gamma-actin	XM_003357928.4	F	GGGCATCCTGACCCTCAAG	89	98.6	[20]
			R	TGTAGAAGGTGTGATGCCAGATCT			
B2M	β-2 microglobulin	NM_213978.1	F	CCCCCGAAGGTTCAGGTT	66	97.463	[20]
			R	GCAGTTCAGGTAATTTGGCTTTC			
GAPDH	Glyceraldehyde 3-phosphate dehydrogenase	NM_001206359.1	F	GGCGTGAACCATGAGAAGTATG	60	101.637	[20]
			R	GGTGCAGGAGGCATTGCT			
HPRT	Hypoxanthine phosphoribosyltransferase	NM_001032376.2	F	AGAAAAGTAAGCAGTCAGTTTCATATCAGT	131	100.767	[20]
			R	ATCTGAACAAGAGAGAAAATACAGTCAATA G			
OAZ1	Ornithine decarboxylase antizyme 1	NM_001122994.1	F	TCGGCTGAATGTAACAGAGGAA	70	99.355	[20]
			R	GAGCCTGGATTGGACGTTTAAA			
				Receptors and transporters involved in SCFA sensing			
FFAR2	Free fatty acid receptor-2	NM_001278758.1	F	CTGCCTGGGATCGTCTGTG	249	104.780	[21]
			R	CATACCCTCGGCCTTCTGG			
FFAR3	Free fatty acid receptor-3	NM_001315601.1	F	GCCCTTGCCCTTCATCTTCT	136	101.724	[21]
			R	CCGGGTCTTGTACCAGAGTG			
HCAR2	Hydroxycarboxylic acid receptor-2	XM_021072989.1	F	AAGGCGATTTCGTAGTTTCCTG	82	99.616	Newly designed
			R	TGGTTCAGTGCTCGCCTTTAT			
HDAC1	Histone deacetylase 1	XM_013999116.2	F	GACCGACTGACGGGAGG	148	100.434	Newly designed
			R	AGTCATGCGGATTCGGTGAG			
TLR2	Toll-Like Receptor-2	NM_213761.1	F	AATAAGTTGAAGACGCTCCCAGAT	97	100.452	[20]
			R	GTTGCTCCTTAGAGAAAGTATTGATCGT			
NLRP3	NLR family pyrin domain containing 3	NM_001256770.2	F	TCAAAGCTCGCTGATGGTGT	110	99.624	Newly designed
			R	GGCACTGTGGTGAAAACTGTC			

EGFR	Epidermal growth factor receptor	NM_214007.1	F<br>R	GCTACGGCAGCTACATAGTC<br>GACAAAGGAATGTCCCTGCAAC	149	100.739	Newly designed
PPARG	Peroxisome proliferator-activated receptor γ	NM_214379.1	F<br>R	CATTCCCGAGAGCTGATCC<br>GGAAGGCTCTTCGTGAGGTT	149	100.264	[21]
SLC5A8 (SMCT1)	Solute Carrier Family 5 Member 8 (Sodium-coupled monocarboxylate transporter 1)	NM_001291414.1	F<br>R	AATCCTCACCTGCTCAGTGC<br>GTAAGCGCAGGCCACAAAAA	172	96.747	Newly designed
SLC5A12 (SMCT2)	Solute Carrier Family 5 Member 12 (Sodium-coupled monocarboxylate transporter 2)	XM_003122908.4	F<br>R	TGGGACACAACTTTCCCTTGG<br>GGAAGGTGGTTCTCCTGTGG	407	97.025	Newly designed
SLC16A1 (MCT1)	Solute Carrier Family 16 Member 1 (Monocarboxylate transporter 1)	AM286425.1	F<br>R	GGTGGAGGTCCTATCAGCAG<br>AAGCAGCCGCCAATAATCAT	74	99.708	[21]
			Tight-junction and adherens junction proteins, defensins and cytokines				
ZO1	Zonula occludens-1	AJ318101.1	F<br>R	TCAAGGTCTGCCGAGACAAC<br>CCAAAGGACTCAGCAGGGTT	75	98.981	Newly designed
OCLN	Occludin	NM_001163647.1	F<br>R	TTGTGGGACAAGGAACGTATTTA<br>TGCCTGCCGACACGTTT	76	98.256	[20]
CLDN1	Claudin 1	NM_001244539.1	F<br>R	TGATGAGGTGCAGAAGATGC<br>CCATGCTGTGGCAACTAAGA	88	98.645	[20]
CLDN4	Claudin 4	NM_001161637.1	F<br>R	CAACTGCGTGGATGATGAGA<br>CCAGGGGATTGTAGAAGTCG	140	98.143	[20]
CDH1	Cadherin-1	NM_001163060.1	F<br>R	TACCTGAACGAGTGGGGCAA<br>CCCATCACATGAGCGTAGGG	118	98.387	[20]
JAML	Junction Adhesion Molecule Like	NM_001244730.1	F<br>R	GAAGATCAGGGAAACTCCGTTCTA<br>AGTCCACCTTTGTCACACGTT	257	100.574	Newly designed
NFKB	Nuclear factor kappa-b	XR_002340533.1	F<br>R	TGGACATTCAACGTCAACAGC<br>AGTGAGCTCAGCATTTCATCCAT	75	100.317	Newly designed
BD3	Prepro-beta-defensin 3	XM_021074698.1	F	AAGTGACCAAGCACGCCTT	70	100.228	Newly designed

			R	GTAGTGGATCCTCATGGCTGG			
EP2C	Epididymis protein 2 splicing variant C	BK005522.1	F	ACTGCTTGTTCTCCAGAGCC	92	101.835	Newly designed
			R	TGGCACAGATGACAAAGCCT			
IL10	Interleukin-10	NM_214041.1	F	GCCTACATGACGATGAAGATGA	112	100.316	[20]
			R	TGAAAGTCTCCAATTTGTATCCTAGAGT			
IL18	Interleukin-18	NM_213997.1	F	AGCTGAAAACGATGAAGACCTG	121	100.852	Newly designed
			R	AAACACGGCTTGATGTCCCT			

References

20. Klinsoda, J.; Vötterl, J.; Zebeli, Q.; Metzler-Zebeli, B.U. Alterations of the viable ileal microbiota of the gut mucosa-lymph node axis in pigs fed phytase and lactic acid-treated cereals. *Appl. Environ. Microbiol.* **2020,** *86*, e02128-19. doi: 10.1128/AEM.02128-19.
21. Newman, M.A.; Petri, R.M.; Grüll, D.; Zebeli, Q.; Metzler-Zebeli, B.U. Transglycosylated starch modulates the gut microbiome and expression of genes related to lipid synthesis in liver and adipose tissue of pigs. *Front. Microbiol*. **2018,** *9*, 224. doi: 10.3389/fmicb.2018.00224.
